# Application of inverse weighting analysis to assess the association of youth perceptions with the age of initiation of tobacco products

**DOI:** 10.3389/fpubh.2024.1203631

**Published:** 2024-02-21

**Authors:** Folefac D. Atem, Meagan A. Bluestein, Baojiang Chen, Melissa B. Harrell, Sarah E. Messiah, Arnold E. Kuk, Adriana Pérez

**Affiliations:** ^1^Department of Biostatistics and Data Science, School of Public Health, The University of Texas Health Science Center at Houston (UTHealth), Houston, TX, United States; ^2^Center for Pediatric Population Health, School of Public Health in Dallas, UT Health Science Center School of Public Health, Dallas, TX, United States; ^3^Michael & Susan Dell Center for Healthy Living, School of Public Health, Austin Campus, The University of Texas Health Science Center at Houston (UTHealth), Austin, TX, United States; ^4^Department of Epidemiology, Human Genetics, and Environmental Sciences, School of Public Health, The University of Texas Health Science Center at Houston (UTHealth), Austin, TX, United States; ^5^Consultant with Litigation Involving the Vaping Industry, Austin, TX, United States

**Keywords:** tobacco, addictiveness, harmfulness, truncation, interval censoring, weighted survival analysis

## Abstract

**Introduction:**

To examine if perceptions of harmfulness and addictiveness of hookah and cigarettes impact the age of initiation of hookah and cigarettes, respectively, among US youth. Youth (12-17 years old) users and never users of hookah and cigarettes during their first wave of PATH participation were analyzed by each tobacco product (TP) independently. The effect of perceptions of (i) harmfulness and (ii) addictiveness at the first wave of PATH participation on the age of initiation of ever use of hookah was estimated using interval-censoring Cox proportional hazards models.

**Methods:**

Users and never users of hookah at their first wave of PATH participation were balanced by multiplying the sampling weight and the 100 balance repeated replicate weights with the inverse probability weight (IPW). The IPW was based on the probability of being a user in their first wave of PATH participation. A Fay’s factor of 0.3 was included for variance estimation. Crude hazard ratios (HR) and 95% confidence intervals (CIs) are reported. A similar process was repeated for cigarettes.

**Results:**

Compared to youth who perceived each TP as “a lot of harm”, youth who reported perceived “some harm” had younger ages of initiation of these tobacco products, HR: 2.53 (95% CI: 2.87-4.34) for hookah and HR: 2.35 (95% CI: 2.10-2.62) for cigarettes. Similarly, youth who perceived each TP as “no/little harm” had an earlier age of initiation of these TPs compared to those who perceived them as “a lot of harm”, with an HR: 2.23 (95% CI: 1.82, 2.71) for hookah and an HR: 1.85 (95% CI: 1.72, 1.98) for cigarettes. Compared to youth who reported each TP as “somewhat/very likely” as their perception of addictiveness, youth who reported “neither likely nor unlikely” and “very/somewhat unlikely” as their perception of addictiveness of hookah had an older age of initiation, with an HR: 0.75 (95% CI: 0.67-0.83) and an HR: 0.55 (95% CI: 0.47, 0.63) respectively.

**Discussion:**

Perceptions of the harmfulness and addictiveness of these tobacco products (TPs) should be addressed in education campaigns for youth to prevent early ages of initiation of cigarettes and hookah.

## Introduction

Many studies have been conducted to assess the prevalence of tobacco product (TP) use in youth, and several studies have suggested risk factors for initiation including previous substance use, lack of awareness, and socioeconomic status (1, 2). Moreover, initiation of TP use is associated with decreased perceptions of harmfulness (3); however, other studies have shown that most youth who initiated cigarette use were aware of the risks associated with these TPs (4). Nonetheless, most of the studies on the impact of harmfulness and addictiveness have focused on the initiation of TP use with binary outcomes (yes/no) (3, 5, 6). Rather than analyzing the initiation as a yes/no answer, monitoring participants over time until some of them experience the event of interest improves precision and decreases bias (7). In a 2022 longitudinal study using Population Assessment of Tobacco and Health (PATH) data, the impact of the perceptions of hookah harmfulness and addictiveness on the age of initiation was examined among never users during their initial participation in the PATH study (8). However, analyses excluded the youth who reported hookah use during their first wave of PATH participation. This type of study design that involves only never users at the outset of the study and follows them prospectively until the occurrence of the event of interest is referred to as an incident cohort design (9). According to Cain et al. (9), excluding these participants may result in biased estimates. In this study, we aimed to incorporate both participants who had already initiated each TP use at their first wave of PATH participation and never users of each TP at that same wave. This approach allowed us to separately investigate their perceptions of hookah and cigarettes regarding their harmfulness and addictiveness at the age of initiation (9). Employing a survival analysis study design, participants were followed until they initiated the TP use (8, 10). To comprehend the impact of the perceptions of harmfulness and addictiveness on the age of initiation for hookah and cigarette use among the youth population, we carefully balanced the effects of users and never users at their first wave of PATH participation through the inverse probability weighting (IPW) method (11, 12). The strength of this approach is that it does not exclude users from the analysis and balances between covariates such as sex and race/ethnicity between users and never users (13, 14).

## Methods

We performed secondary data analysis using the PATH data, which is a longitudinal study of tobacco use in the US (15). This study began during 2013–2014 with annual samples collected until 2017; when samples were collected every 2 years a complex stratified area probability sampling method was employed to generate a nationally representative sample of youth and adults in the US (15, 16). We included youth (aged 12–17 years) data from waves 1–4 (wave 1: 2013–2014, wave 2: 2014–2015, wave 3: 2015–2016, and wave 4: 2016–2017). Shadow youth participants, that is, youth aged 9–11 years at wave 1, entered the study once they turned 12 at wave 2 and wave 3, respectively. All youth who turned 18 years in the subsequent waves were invited to participate in the adult study, and their follow-up data as adults were included in the analyses (15, 16). The institutional review board approval for this study was obtained from the Committee for the Protection of Human Subjects at the University of Texas Health Science Center at Houston (protocol number HSC-SPH-17-0368).

### How PATH measures TPs?

In the first wave of participation (waves 1–4), participants were asked the following question: “Have you ever smoked tobacco in a hookah, even one or two puffs?,” and the response options were “yes,” “no,” and “I do not know” (15). In waves 1–4, using PATH, youth respondents were asked the following question: “Have you ever smoked a cigarette, even one or two puffs?,” and the response options were “yes,” “no,” and “I do not know (15, 16). These analyses were limited to participants that responded “yes” or “no.”

### Interval-censored outcome: ever age of initiation of TP

The restricted-use PATH data do not provide the date of birth information for youth participants; however, it does provide the participants’ age in years at each wave and the number of weeks between the waves. For each participant, we created a lower and an upper age bound for each TP outcome. At the first wave of PATH participation, youths were asked the following question: “Have you ever smoked tobacco in a hookah, even one or two puffs?,” and the response options were “yes” and “no.” Those who answered “yes” were asked another question: “Age when first tried smoking a hookah, even one or two puffs?” This recalled age of initiation as indicated by the youth is what we labeled “recalled age.” Similar questions were asked about cigarettes, separately.

The lower age bound for the youth who indicated the use of TPs at their first wave of PATH participation was their recalled age of initiation, while the upper age bound was the age at the first wave of PATH participation (17).

For never users of each TP at their first wave of PATH participation, the lower age bound was the participants’ age at the first wave of PATH participation plus the number of weeks between the first wave and the last wave the participant reported non-use of each TP. For those who initiated the TP after the first wave of PATH participation, the upper age bound was the age at the lower bound plus the number of weeks between the lower bound and the first wave the participant reported the initiation of each TP (18). Never users during the study period were considered right-censored, that is, their upper bound age of initiation was unbounded (19).

### Exposures: perceptions of harmfulness and of addictiveness of each TP

PATH assessed perceptions of harmfulness and addictiveness of hookah and cigarettes individually for youth in their first wave of PATH participation (waves 1–3). The perceptions of harmfulness of cigarettes and hookah in waves 1–3 were only assessed among the youth participants who had heard of each TP at each wave. Since all users of a TP at their first wave of PATH participation must have heard of the TP, never users, those who had not heard of each TP, were excluded. The following question was used to measure the perceptions of harmfulness: “How much do you think people harm themselves when they smoke/use [TP]?” (16). The response options included (i) “no harm”; (ii) “little harm”; (iii) “some harm”; (iv) “a lot of harm”; (v) “do not know”; and (vi) “refused.” These responses were recoded as “no/little harm,” “some harm,” and “a lot of harm.” The participants who answered “do not know” and “refused” were not included in the analysis.

Similarly, the perceptions of addictiveness of each TP were assessed at the first wave of PATH participation, using the questions, “How likely is someone to become addicted to shisha or hookah tobacco?” or “How likely is someone to become addicted to cigarettes?” (16). The response options included (i) “very unlikely”; (ii) “somewhat unlikely”; (iii) “neither likely nor unlikely”; (iv) “somewhat likely”; (v) “very likely”; (vi) “do not know”; and (vii) “refused.” We excluded those who responded with “do not know” and “refused” from our analyses. The variables were recoded as follows: “very/somewhat unlikely,” “neither likely nor unlikely,” and “somewhat/very likely.” Similarly, the youth who had not heard of each TP were excluded from the analyses.

### Covariates: Sex and race/ethnicity

Sex was classified as female or male, and this variable was imputed by PATH using the household information at wave 1 (16). Race was assessed as white, Black, Asian, and other (including multi-racial). Ethnicity was categorized as either Hispanic or non-Hispanic. In our analyses, compared to the previous publications, we categorized race/ethnicity into four categories, with non-Hispanic white as the reference: non-Hispanic white, Hispanic, non-Hispanic Black, and non-Hispanic other (Asian, multi-race, and other races) (19, 20, 21).

### Statistical analysis

A two-phase secondary analysis for understanding the perceptions of harmfulness and addictiveness at the age of initiation of hookah or cigarette use was conducted among the youth who participated in PATH (aged 12–17 years) at their first wave of PATH participation (waves 1–3) (16). In the first phase, we employed the IPW method, considering the probability of being a user, to balance the confounding effects related to sex and race/ethnicity between users and never users of each TP at their first wave of PATH participation (12). The primary goal of IPW is to create a pseudo-population where the distribution of sex and race/ethnicity is balanced across TP users and never users at their first wave of PATH participation, similar to what might be observed in a randomized controlled trial. This weighting technique helps in mitigating the biases that could arise due to sex and race/ethnicity or uneven distribution of characteristics among TP users and never users at their first wave of PATH participation, thereby allowing for more accurate estimations of treatment effects or associations between variables.

The IPW technique uses propensity score to balance the characteristics of users and never users of each TP (hookah and cigarettes) by weighing each individual in the analysis (22).

Specifically, we developed a weight known as IPW by fitting a logistic regression with the binary indicator variable (yes/no) for users versus never users at the first wave of PATH participation for each TP with sex and race/ethnicity as the covariates (12, 23).

Thus, the propensity score, defined as p(K) = Pr(*Z* = 1 | K), is the conditional probability of being a user of hookah (yes/no) at the first wave of PATH participation, that is, *Z* = 1 for users of hookah at their first wave of PATH participation and *Z* = 0 for never users of hookah at their first wave of PATH participation. K refers to the measured covariates, that is, sex and race/ethnicity. The p(K) is estimated using a logistic regression for the probability of being a user of hookah at the first wave of PATH participation with sex and race/ethnicity as covariates, and its estimate (output) represented the predictive value from this model. Then, weights are assigned for users and never users of each TP by exemplifying the case for hookah. Hookah users at their first wave of PATH participation were assigned a weight: w(K) = 1/p(K), whereas never users of hookah were assigned a weight: w(K) = 1/1-p(K) (11, 12, 23). Weight assignment was repeated for cigarette users and non-users at the first wave of PATH participation. Next, we created a new set of composite weights by (1) multiplying the IPW with the PATH sampling weight and (2) multiplying the IPW with the 100 balance replicate weights (24, 25). This new set of composite weights is expected to minimize the bias due to the lack of randomization in being a user or never user at the first wave of PATH participation (24). Differences in perceptions of harmfulness and addictiveness were explored by fitting composite weighted Cox proportional hazards regression models to interval-censored data with a constant baseline hazard function, which represents the instantaneous rate of initiation of a TP within the interval. Hazard ratios (HR), serving as indicators for the likelihood of TP initiation between groups, were estimated, and their respective 95% confidence intervals (CI) were reported (26, 27). The hazard function and non-parametric estimators for each significant level by perceptions of harmfulness and addictiveness, separately from the Cox proportional hazards regression models, were estimated (28–30). A type I error level of 0.05 was used. For each TP, Statistical Analysis System (SAS version 9.4) macros were developed.

## Results

Weighted summary statistics for demographic characteristics for PATH youth (aged 12–17 years at their first wave of PATH participation) are shown in Table 1. The sample size for the users and never users (waves 1–3) for each of the TPs was as follows: 16,564 youth (representing 30,85,8,246 US youth) for hookah, and 17,689 youth (representing 32,982,770 US youth) for cigarettes. Among these youth, 5.9% ever used hookah and 10.7% ever used cigarettes by their first wave of PATH participation. The average age of participants in their first wave of PATH participation was 13.9 years for these TPs. The distribution of sex was almost evenly distributed, with girls accounting for approximately 49.1% of the hookah sample and 48.8% of the cigarette sample. The racial/ethnic distribution among various TP usage was notably consistent, with approximately 54% being non-Hispanic white, 23% Hispanic, 13% non-Hispanic Black, and 10% falling into the non-Hispanic other category. The majority of youth perceived both hookah and cigarettes as harmful, with 52.3% reporting “a lot of harm” for hookah and 83.3% for cigarettes. Additionally, a considerable percentage of youth perceived hookah (75.3%) and cigarettes (81.5%) to be “somewhat/ very likely” in terms of addictiveness.

**Table 1:** Demographic characteristics of users and never users of each TP in PATH USA youth (aged 12–17 years).

Variables		Hookah			Cigarettes		
		*n* = 16,564	*N* = 30,858,246	Weighted % (SE)	*n* = 17,689	*N* = 32,989,770	Weighted % (SE)
Wave of entry into path	Wave 1 (2013–2014)	12,552	22,781,268	73.8 (0.19)	13,583	24,730,664	75.0 (0.15)
	Wave 2 (2014–2015)	2,000	3,937,998	12.8 (0.14)	2,080	4,092,448	12.4 (0.13)
	Wave 3 (2015–2016)	2,012	4,138,980	13.4 (0.21)	2,026	4,166,676	12.6 (0.19)
Proportion of users at entry		996	1,834,228	5.9 (0.28)	1,940	3,521,686	10.7 (0.33)
Age at entry into the study (SE)	Weighted mean (SE)	*n* = 16,564	13.9	0.01	*n* = 17,689	13.9	0.01
Sex	Female	8,105	15,146,244	49.1 (0.12)	8,597	16,073,450	48.8 (0.08)
	Male	8,451	15,693,004	50.9 (0.12)	9,083	16,896,371	51.2 (0.08)
	Missing	8			9		
Race/ethnicity	Non-Hispanic white	7,957	16,519,908	53.7 (0.17)	8,526	17,685,987	53.7 (0.12)
	Hispanic	4,815	7,159,820	23.3 (0.14)	5,076	7,544,231	22.9 (0.11)
	Non-Hispanic Black	2,183	4,089,071	13.3 (0.11)	2,376	4,462,032	13.6 (0.07)
	Non-Hispanic others^Ϯ^	1,564	3,009,399	9.8 (0.12)	1,661	3,213,348	9.8 (0.09)
	Missing	45			47		
Perception of harmfulness	No/little harm	2,855	5,256,695	17.0 (0.43)	522	912,600	2.8 (0.14)
	Some harm	5,152	9,462,627	30.7 (0.45)	2,520	4,579,952	13.9 (0.33)
	A lot of harm	8,557	16,138,924	52.3 (0.63)	14,647	27,497,218	83.3 (0.38)
Perception of addictiveness	Very/somewhat unlikely	1,769	3,262,520	10.6 (0.27)	1,883	3,390,306	10.3 (0.25)
	Neither likely nor unlikely	2,368	4,344,067	14.1 (0.34)	1,499	2,715,850	8.2 (0.22)
	Somewhat/very likely	12,427	23,251,658	75.3 (0.44)	14,307	26,883,614	81.5 (0.34)

*PATH Restricted file received disclosure to publish: 22 April 2021. United States Department of Health and Human Services. National Institutes of Health. National Institute on Drug Abuse, and United States Department of Health and Human Services. Food and Drug Administration. Center for Tobacco Products. Population Assessment of Tobacco and Health (PATH) Study [United States] Restricted-Use Files. ICPSR 36231-v13. AnnArbor, MI: Inter-university Consortium for Political and Social Research [distributor], 5 November 2019. https://doi.org/10.3886/ICPSR36231.v23.

^Ϯ^Non-Hispanic others include Asian, multi-race, etc.

In Table 2, we present the adjusted hazard ratios and their 95% CIs exploring the impact of the perceptions of harmfulness and addictiveness on the age of initiation of each TP using the composite weight and the 100 composite BRR weights. Adjusted hazard ratios are balanced by sex and race/ethnicity. The findings revealed distinct patterns regarding perceptions of harm and addictiveness influencing the initiation of hookah and cigarette use among youth. Youth perceiving hookah as having “no/little harm” showed a strikingly higher likelihood (123%, HR, 2.23, 95% CIs, 1.82–2.73) of being more prone to initiate hookah use earlier compared to those perceiving it as having “a lot of harm.” Similarly, those perceiving hookah as having “some harm” were 253% (HR: 3.53, 95% CI: 2.87–4.34) more likely to initiate use at younger ages than those perceiving “a lot of harm.”

**Table 2:** Hazard ratios (and 95% confidence intervals) for the association between perceptions of harmfulness and addictiveness and the age of initiation of hookah and cigarette use.

Variable	Hookah	Cigarettes
	*n* = 16,564; *N* = 30,858,246	*n* = 17,689; *N* = 32,989,770
Perception of harmfulness	Adjusted hazard ratios	Adjusted hazard ratios
A lot of harm	1.00	1.00
Some harm	3.53 (2.87, 4.34)	2.35 (2.10, 2.62)
No/little harm	2.23 (1.82, 2.71)	1.85 (1.72, 1.98)

Perception of addictiveness	Adjusted hazard ratios	Adjusted hazard ratios
Somewhat/very likely	1.00	1.00
Neither likely nor unlikely	0.75 (0.67, 0.83)	1.08 (0.94, 1.23)
Very/somewhat unlikely	0.55 (0.47, 0.63)	0.98 (0.88, 1.10)

*PATH Restricted file received disclosure to publish: 22 April 2021. United States Department of Health and Human Services. National Institutes of Health. National Institute on Drug Abuse, and United States Department of Health and Human Services. Food and Drug Administration. Center for Tobacco Products. Population Assessment of Tobacco and Health (PATH) Study [United States] Restricted-Use Files. ICPSR 36231-v13. AnnArbor, MI: Inter-university Consortium for Political and Social Research [distributor], 5 November 2019. https://doi.org/10.3886/ICPSR36231.v23.

Regarding the addictiveness perceptions, the youth who reported feeling “very/somewhat unlikely” were 45% (HR: 0.55, 95% CI: 0.47–0.63) less likely to initiate hookah use early compared to those feeling “somewhat/very likely.” Meanwhile, those with “neither likely nor unlikely” perceptions were 25% (HR: 0.75, 95% CI: 0.67–0.83) less inclined to initiate hookah use at a younger age compared to those perceiving addictiveness as “somewhat/very likely.” Concerning cigarettes, youth perceiving “no/little harm” and “some harm” were notably 85% higher for early age of initiation (HR: 1.85, 95% CI: 1.72-1.98) and 135% (HR: 2.35, 95% CI: 2.10-2.62) respectively, as compared to those perceiving cigarettes as having “a lot of harm”.

Table 3 shows the hazard function and its 95% CI of ever use of each of these TPs stratified by three categorized classes of the perceptions of harmfulness. For all three TPs, initiation increases as the perceptions of harmfulness decrease from “a lot of harm” to “no/little harm.” By the age of 17 years, the proportion of youth who perceived “a lot of harm” from hookah and initiated it was 3.4% (95% CI, 2.8–4.1), those who perceived “some harm” and initiated it was 10.8% (95% CI, 9.1–11.8), and those who perceived “no/little harm” and initiated it was 27.7% (95% CI, 25.0–30.3). Moreover, by the age of 17 years, the proportion of youth who perceived “a lot of harm” in cigarettes and initiated them was 22.9% (95% CI, 20.2–25.6), those who perceived “some harm” and initiated them was 40.0% (95% CI, 33.3–45.7), and those who perceived “no/little harm” and initiated them was 42.1% (95% CI, 36.1–48.1).

**Table 3:** Estimated hazard function (95% confidence interval) of the age of initiation of ever TP use outcomes by perceptions of harmfulness of PATH USA youth.

	Hookah	Cigarettes
	*n* = 16,564; *N* = 30,858,246	*n* = 17,689; *N* = 32,989,770
No/little harm		
Age of initiation	Hazard function	Hazard function
12	0.5% (0.2, 0.9)	16.8% (11.4, 22.2)
13	1.7% (1.2, 2.3)	16.8% (11.5, 22.0)
14	3.9% (3.0, 4.8)	22.7% (14.1, 31.4)
15	8.3% (7.1, 9.5)	29.9% (25.6, 34.2)
16	17.2% (15.2, 19.1)	34.4% (29.8, 39.0)
17	27.7% (25.0, 30.3)	42.1% (36.1, 48.1)
18	NA^Ϯ^	54.0% (43.0, 65.0)
19	NA	54.0% (47.3, 60.8)
20	NA	70.1% (42.8, 98.3)

Some harm		
Age of initiation	Hazard function	Hazard function
12	0.3% (0.1, 0.4)	8.7% (6.8, 10.6)
13	0.4% (0.2, 0.7)	9.4% (7.5, 11.4)
14	0.9% (0.6, 1.2)	14.6% (12.5, 16.7)
15	2.5% (1.9, 3.1)	25.9% (18.3, 33.5)
16	5.8% (4.9, 6.6)	30.0% (25.2, 34.9)
17	10.4% (9.1, 11.8)	40.0% (33.3, 45.7)
18	NA	42.0% (37.8, 46.1)
19	NA	50.0% (45.8, 54.1)
20	NA	54.1% (47.2, 61.0)

A lot of harm		
Age of initiation	Hazard function	Hazard function
12	0.0% (0.0, 0.1)	4.2% (3.6, 4.7)
13	0.1% (0.0, 0.2)	4.6% (4.1, 5.2)
14	0.3% (0.1, 0.5)	8.8% (6.5, 11.1)
15	0.6% (0.4, 0.9)	13.1% (11.0, 15.2)
16	1.8% (1.3, 2.2)	18.1% (16.6, 19.5)
17	3.4% (2.8, 4.1)	22.9% (20.2, 25.6)
18	NA	31.3% (29.3, 33.2)
19	NA	32.3% (30.0, 34.5)
20	NA	36.9% (34.7, 39.1)

*PATH Restricted file received disclosure to publish: 22 April 2021. United States Department of Health and Human Services. National Institutes of Health. National Institute on Drug Abuse, and United States Department of Health and Human Services. Food and Drug Administration. Center for Tobacco Products. Population Assessment of Tobacco and Health (PATH) Study [United States] Restricted-Use Files. ICPSR 36231-v13. AnnArbor, MI: Inter-university Consortium for Political and Social Research [distributor], 5 November 2019. https://doi.org/10.3886/ICPSR36231.v23.

^Ϯ^NA means not enough samples for estimation.

Table 4 shows the hazard functions and its 95% CIs of ever use of each of these TPs stratified by three categorized classes of the perceptions of addictiveness. The results limited to the strata that are significantly different from the reference class “somewhat/very likely” perception of addictiveness were presented. Our results showed that, by the same age, the proportion of initiation of hookah increases as the perception of addictiveness decreases from “somewhat/very likely” to “very/somewhat unlikely.” For example, by the age of 17 years, 5.2% (95% CI: 4.4–6.0) of youth were estimated to initiate hookah among those who reported “somewhat/very likely” perceptions of addictiveness for hookah, 16.2% (95% CI: 14.0–18.4) of youth were estimated to initiate hookah among those who reported “neither likely nor unlikely” perceptions of addictiveness, and 38.8% (95% CI: 30.5–37.1) of youth were estimated to initiate hookah among those who reported “very/somewhat unlikely” perceptions of addictiveness.

**Table 4:** Estimated hazard function (95% confidence interval) of the age of initiation of ever TP use outcomes by perceptions of addictiveness of PATH USA youth.

	Hookah	Cigarettes
	*n* = 16,564; *N* = 30,858,246	*n* = 17,689; *N* = 32,989,770
Very/somewhat unlikely		
Age of initiation	Hazard function	Hazard function
12	0.8% (0.3, 1.3)	6.1% (4.3, 8.0)
13	1.6% (1.0, 2.2)	6.9% (5.3, 8.5)
14	3.3% (2.3, 4.3)	9.3% (7.3, 11.2)
15	9.2% (7.7, 10.7)	12.9% (10.8, 15.0)
16	20.5% (18.2, 22.8)	22.5% (16.6, 28.4)
17	33.8% (30.5, 37.1)	22.9% (18.2, 27.4)
18	NA^Ϯ^	33.4% (22.1, 44.7)
19	NA	34.8% (30.2, 39.4)
20	NA	46.5% (35.6, 57.5)

Neither likely nor unlikely		
Age of initiation	Hazard function	Hazard function
12	0.2% (0.0, 0.3)	
13	0.8% (0.3, 1.2)	
14	2.0% (1.2, 2.7)	
15	4.3% (3.2, 5.3)	
16	9.3% (7.6, 11.1)	
17	16.2% (14.0, 18.4)	
18	NA	
19	NA	
20	NA	

Somewhat/very likely		
Age of initiation	Hazard function	Hazard function
12	0.1% (0.0, 0.2)	4.9% (4.2, 5.5)
13	0.3% (0.2, 0.4)	5.4% (4.8, 6.1)
14	0.7% (0.5, 0.9)	10.3% (7.6, 12.9)
15	1.5% (1.1, 1.9)	15.9% (13.9, 18.0)
16	3.5% (2.9, 4.0)	19.9% (17.4, 22.3)
17	5.2% (4.4, 6.0)	26.1% (23.5, 28.7)
18	NA	33.4% (30.0, 37.1)
19	NA	35.0% (33.0, 37.0)
20	NA	39.4% (37.2, 41.6)

*PATH Restricted file received disclosure to publish: 22 April 2021. United States Department of Health and Human Services. National Institutes of Health. National Institute on Drug Abuse, and United States Department of Health and Human Services. Food and Drug Administration. Center for Tobacco Products. Population Assessment of Tobacco and Health (PATH) Study [United States] Restricted-Use Files. ICPSR 36231-v13. AnnArbor, MI: Inter-university Consortium for Political and Social Research [distributor], 5 November 2019. https://doi.org/10.3886/ICPSR36231.v23.

^Ϯ^NA means not enough samples for estimation.

## Discussion

In this study, we examined the association between the youth perceptions of harmfulness and addictiveness and the age of initiation of ever use of hookah and cigarettes. These two TPs reported more than 5% ever use at the first wave of PATH participation. Each TP product was examined independently to understand how youth’s perceptions of harmfulness and addictiveness influence the age of initiation of each of these TPs. This study differs significantly from the two previous studies (8, 10) in two ways. First, the dataset analyzed by Chen et al. (10) and Kuk et al. (8) was limited to non-users of TPs at their first wave of PATH participation, while we included both users and non-users of each TP at the first wave of PATH participation, separately, in this study. Second, in the previous two studies, the analyses took advantage of the PATH sampling weights and the 100 balance replicate weights; however, in this study, we created composite weights by multiplying the IPW with the PATH sampling weight and multiplying the IPW with the 100 balance replicate weights. In our inverse probability approach, we aimed to achieve equilibrium in sex and race/ethnicity distributions. This balancing act mirrors block randomization based on sex and race/ethnicity. The goal is to ensure that any observed disparities between groups are not attributed to differences in sex or race/ethnicity but instead reflect the impact of other factors under examination.

The findings from the previous studies indicated that, when youth perceived these tobacco products as less harmful, they tended to initiate their use at earlier ages. Our results support this trend with a nuanced twist: the likelihood of initiating ever use of cigarette and hookah at younger ages follows the order of “some harm” > “no/little harm” > “a lot of harm.”

Moreover, a previous study revealed that a lower perceived risk was linked to the likelihood of ever using hookah (31). Our study, in conjunction with this research, highlights the potential impact of educating youth about the potential harmfulness and addictiveness of hookah, which could significantly decrease its consumption. Overall, the analysis results suggest that educating these young individuals about the potential risks associated with these tobacco products might prove beneficial in altering their perceptions of them.

This nationally representative US sample, combined with a causal analytical approach that balanced users and non-users during their initial participation in the PATH study, offered valuable insights into how perceptions of harmfulness and addictiveness influence the age of hookah and cigarette initiation. Moreover, our longitudinal study design strengthens the validity of findings, illustrating a robust and consistent relationship between risk perceptions and the age at which individuals initiate hookah and cigarette use.

A potential limitation in the design of this study is that the perceptions of harmfulness and addictiveness were recorded at the first wave of PATH participation, where users of each TP were recorded after the initiation, while never users of each TP were recorded before the initiation. Despite these measurement issues, we do not think this will have a tremendous effect on our results since we assumed the exposure does not change over time in the model (32, 33). Another potential drawback is with the recalled age of initiation for users at their first wave of PATH participation because these users might not precisely know when they initiated the TP. However, in one of our previous publications, we performed a sensitivity analysis where we replaced the recalled age of initiation with a uniform six “6” as the lower age bound under the assumption that the participants were at least 6 years old when they initiated each of these products. The results showed insignificant differences using the recalled age and the uniform six “6” as the lower age bound for these TPs (17), indicating that recall bias was minimal.

## Conclusion

Our findings highlight the importance of the perceptions of harmfulness and addictiveness in the age of initiation of hookah and cigarettes. The perceptions of harmfulness and addictiveness of these TPs should be addressed in education campaigns for youth to prevent earlier ages of initiation of these products.

## Data availability statement

The datasets presented in this article are not readily available because we used the restricted data from Population Assessment of Tobacco and Health (PATH) study, 2013–2017. Any researcher could assess the data after completing the necessary paperwork. Requests to access the datasets should be directed to nahdap-vde-support@umich.edu.

## Author contributions

FA, MH, SM and AP wrote the protocol. BC, MB, AK, and AP conducted literature search and data compilation. FA analyzed the data and wrote the first draft of the manuscript. MH, SM, and AP supervised and redrafted the manuscript. FA and AP assembled the final draft and circulated it to all the authors who read and agreed on the final submitted draft.
